# Factors predicting parametrial invasion in patients with early-stage cervical carcinomas

**DOI:** 10.1371/journal.pone.0204950

**Published:** 2018-10-18

**Authors:** Heng-Cheng Hsu, Yi-Jou Tai, Yu-Li Chen, Ying-Cheng Chiang, Chi-An Chen, Wen-Fang Cheng

**Affiliations:** 1 Department of Obstetrics and Gynecology, College of Medicine, National Taiwan University, Taipei, Taiwan; 2 Department of Obstetrics and Gynecology, National Taiwan University Hospital Hsin-Chu Branch, Hsin-Chu City, Taiwan; 3 Graduate Institute of Clinical Medicine, College of Medicine, National Taiwan University, Taipei, Taiwan; 4 Graduate Institute of Anatomy and Cell Biology, College of Medicine, National Taiwan University, Taipei, Taiwan; 5 Graduate Institute of Oncology, College of Medicine, National Taiwan University, Taipei, Taiwan; Tata Memorial Centre, INDIA

## Abstract

We aimed to identify factors predicting parametrial invasion in early-stage cervical cancer patients undergoing radical hysterectomy. We recruited women with invasive cervical cancer who underwent radical hysterectomy at a single medical institute from 2000–2011. The clinical and pathological characteristics and outcomes were retrospectively recorded, and the risk factors for parametrial invasion were analyzed. We enrolled 339 patients, including 7 with stage IA1 carcinomas, 10 with stage IA2, 266 with stage IB1, 39 with stage IB2, 14 with stage IIA1, and 3 with stage IIA2. The majority (237/339, 69.9%) had squamous cell carcinoma, while 32 (12.4%) had parametrial invasion. The 16 patients with stage IB1 tumors and parametrial invasion were older (55.9±9.5vs. 49.0±9.9 years, p = 0.005, Mann-Whitney U test), and had deeper cervical stromal invasion (9.59±4.87 vs. 7.47±5.48 mm, p = 0.048, Mann-Whitney U test), larger tumor size (2.32±1.15 vs. 1.74±1.14cm, p = 0.043, Mann-Whitney U test), higher incidences of lymphovascular space invasion (87.5% vs. 28.8%, p<0.001, chi-square test), and greater lymph node metastasis (68.8% vs. 10.8%, p<0.001, chi-square test) than the 260 patients without parametrial invasion. Among the patients with stage IB1 tumor size >2 cm,10% had parametrial invasion and 24.2% had lymph node metastasis compared with only 4% and 9.4% of stage IB1 patients with a tumor size <2 cm, respectively. Only one (0.9%) of the 109 patients aged less than 50 years had parametrial invasion compared with 6 (9.7%) of the 62 patients aged over 50 years. Patients with stage IA2 and IB1 tumors <2 cm may not need radical hysterectomy owing to the low incidence of parametrial invasion.

## Introduction

Cervical cancer has the fourth highest incidence and mortality rate among cancer in women [1]. The primary treatments for early-stage cervical cancer (stages I and IIA) include radiation and surgery [2–6]. The surgical intervention for early-stage cervical cancer is radical hysterectomy, except for stage IA1 [3]. However, while the prognosis after radical hysterectomy is good, the operation includes parametrectomy, which damages the autonomic nerves traversing through the paracervical region. These nerves include the sympathetic fibers from the hypogastric nerve and parasympathetic fibers from the pelvic splanchnic and inferior hypogastric plexus [7]. As a result, the associated long-term morbidity often greatly reduces the patient’s quality of life, leading to lower urinary tract and sexual dysfunction. Some investigators have attempted to develop nerve-sparing procedures during radical hysterectomy to preserve nerve and bladder function [8, 9]. However, such techniques are not yet standardized, and the oncologic therapeutic effect is unclear.

Many investigators sought to find a subgroup of early-stage cervical cancer patients with a low incidence of parametrial metastasis who might not need parametrectomy during the hysterectomy [10–14] to improve the morbidities of radical surgery. Factors such as a tumor size <2 cm, depth of invasion <10 mm, absence of lymphovascular space invasion (LVSI), and negative pelvic lymph nodes are associated with a low incidence of parametrial invasion [15]. However, no definitive risk factors have been identified. Thus, we conducted this retrospective study to ascertain whether certain specific factors could help predict the possibility of parametrial invasion in early cervical cancer. This may preclude unnecessary parametrectomy, lower the incidences of morbidity and mortality, and improve the quality of life of early-stage cervical cancer patients.

## Materials and methods

### Patients

This study was approved by the Research Ethics Committee at the National Taiwan University Hospital (201801122RIN). All of the patients’ data were fully anonymized before we accessed them and the Research Ethics Committee waived the requirement for informed consent. The medical records in the National Taiwan University Hospital database of women diagnosed with invasive cervical cancer between the 1st of January 2000 and the 31st of December 2011 were retrospectively reviewed. The study protocol was approved by the Institutional Review Board of the hospital. Patients were eligible if they met the following criteria: (1) they were diagnosed at stage IA2 to IIA2; (2) they were treated with a class II or III radical hysterectomy [16]; (3) they were treated with pelvic lymphadenectomy; (4) the histologic data were reviewed by proficient pathologists specializing in gynecologic oncology; (5) there were sufficient clinico-pathological and survival data regarding disease prognosis; and (6) they were not treated with neoadjuvant chemotherapy or radiotherapy. All of the patients were clinically staged according to the criteria of the International Federation of Gynecology and Obstetrics (FIGO) [17]. Histological types were defined according to the World Health Organization classification [18].

### Data collection

Demographic and clinical data, including the age at diagnosis, tumor histology and grade, FIGO stage, type of radical hysterectomy, type of adjuvant chemotherapy and/or radiotherapy, and follow-up data, were extracted from individual medical records and stored in a database. Histological grading of squamous cell carcinomas was based on the modified system proposed by Broders [19], and the grading of adenocarcinomas was based on the architectural features, which closely resemble those of endometrial adenocarcinomas [20]. Disease staging was based on the FIGO classification [17]. Radical hysterectomies were classified as class I to V [16].

Periodic examinations during follow-up comprised history-taking, pelvic and rectal examinations, and lymph node palpation every 3 months for 2 years and then every 6 months thereafter. A Papanicolaou (Pap) smear of the vaginal cuff was done at each visit. A chest X-ray was taken at 6-month intervals for 2 years and yearly thereafter. Tumor markers, including squamous cell carcinoma-associated antigen and carcinoma antigen125, were checked every 3 months for 2 years and then every 6 months thereafter. A complete blood count and biochemistry profile were performed annually. Computerized tomography (CT) was performed when recurrence was suspected. Recurrence was defined according to an abnormal result on a CT scan, aspiration cytology from ascites, or tissue from a biopsy, when possible. Recurrence was categorized into vaginal, local, or distant. Local recurrences included those at the pelvic cavity. Distant recurrences were defined as recurrences in sites other than the vagina or pelvis, such as paraaortic lymphadenopathy and spine, liver, chest, brain, skin, neck, or pleural effusion metastasis.

### Statistical analysis

Statistical analyses were performed using SPSS version 20.0 for Windows (SPSS Inc., Chicago, IL). Categorical and continuous variables were evaluated with the non-parametric chi-square test, the Kruskal-Wallis test, and the Mann-Whitney U test. All statistical tests were two-sided, and a p value <0.05 was considered statistically significant.

## Results

A total of 339 patients with invasive cervical cancer between 2000 and 2011 underwent class II-III radical hysterectomy and met the inclusion criteria. The mean age of the patients was 49.4 years (range, 21–77 years). The median follow-up time was 85.1 months (range, 0–168 months). Of the 339 patients undergoing radical hysterectomy, 7 had stage IA1 carcinomas, 10 had stage IA2, 266 (78.2%) had stage IB1, 39 had stage IB2, 14 had stage IIA1, and 3 had stage IIA2. The 276 cervical cancer patients, including 10 patients with stage IA2 tumors and 266 with stage IB1, were recruited for further analysis.

### Clinico-pathological characteristics

The clinico-pathological characteristics of the 276 cervical carcinoma patients at stages IA2 and IB1 undergoing radical hysterectomy and bilateral pelvic lymph node dissection are presented in Table 1. The patients were divided into 3 groups according to tumor stage and size. There were no statistical differences in terms of age, gravida, parity, menopausal status, histologic type, or the number of lymph node dissections between these 3 groups. The group with stage IB1 tumors >2 cm had statistically deeper cervical stromal invasion (9.99±5.04 mm, p<0.001, Kruskal-Wallis test), higher incidences of LVSI (45.3%, p = 0.002, chi-square test), and greater lymph node metastasis (24.2%, p = 0.002, chi-square test) compared to the other 2 groups (stage IA2 and stage IB tumors ≤2 cm).The group with stage IB1 tumors >2 cm showed a trend toward a higher incidence of parametrial invasion (9.5%, p = 0.14, chi-square test) compared with the other two groups (stage IA2 tumor, 0%;stage IB1 tumor ≤2 cm, 4%), though it did not reach statistical significance.

**Table 1:** Clinico-pathologic characteristics of 276 patients with stage IA2 and IB1 cervical carcinomas undergoing radical hysterectomy and bilateral pelvic lymph node dissection.

Characteristics	IA2 (%)	IB1 (%)	IB1 (%)	p
		**(≤2 cm)**	**(>2 cm)**	
**Patient number**	10	171	95	
**Age (years)**				
Mean±SD	52.7±12.3	49.1±10.0	49.6±9.8	0.52^a^
(Min-max)	(32–75)	(21–73)	(29–69)	
**Gravida** (Mean±SD)	4.3±2.4	3.6±1.8	3.8±2.0	0.63^a^
**Parity** (Mean±SD)	3.3±1.8	2.6±1.4	2.6±1.4	0.33^a^
**Menopause**				
No	7 (70.0)	102 (60.4)	53(58.8)	0.79^b^
Yes	3 (30.0)	67 (39.6.)	38(41.2)	
**Histologic type**				
SCC	8 (80.0)	113 (66.1)	71 (74.7)	0.70^b^
ADC	2 (20.0)	45 (26.3)	16 (16.8)	
ASC	0	7 (4.1)	5 (5.3)	
NEC	0	3 (1.8)	1 (1.1)	
Others*	0	3 (1.8)	2 (2.1)	
**Tumor size** (cm)(Mean±SD)	0.52±0.57	1.09±0.74	2.98±0.51	<0.001^a^
**DOI**^#^ (mm)(Mean±SD)	3.45±1.07	6.63±5.51	9.99±5.04	<0.001^a^
**LVSI**^&^				
No	9 (90%)	126 (73.7)	52 (57.7)	0.002^b^
Yes	1 (10%)	45 (26.3)	43 (45.3)	
**Parametrial invasion**				
No	10 (100)	164 (96.0)	86 (90.5)	0.14^b^
Yes	0 (0)	7 (4.0)	9 (9.5)	
**Number of lymph node**				
**dissection**				
Mean±SD	24.0± 5.4	22.8±9.0	24.7±10.7	0.38^a^
(Min-max)	19–34	4–58	7–61	
**Lymph node metastasis**				
No	10 (100)	155 (90.6)	72 (75.8)	0.002^b^
Yes	0	16 (9.4)	23 (24.2)	
<3	0	11	13	
≥3	0	5	10	

Min: minimum, max: maximum, SD: standard deviation, %: percentage, SCC: squamous cell carcinoma, ADC: adenocarcinoma, ASC: adenosquamous carcinoma, NEC: neuroendocrine carcinoma, DOI: depth of cervical stromal invasion

*: including two adenocarcinoma with focal neuroendocrine differentiation, two clear cell carcinoma, and one undifferentiated carcinoma

#: depth of cervical stromal invasion

&: lymphovascular space invasion

^a^: by Kruskal-Wallis test

^b^:by Chi-square test.

The clinico-pathologic characteristics of the 276 patients at stages IA2 and IB1 with or without parametrial invasion are presented in Table 2. Sixteen (5.8%) of the 276 patients showed parametrial invasion. Overall, the parametrial invasion group was older (55.94±9.49 vs. 48.95±9.91 years, p = 0.005, Mann-Whitney U test),had deeper cervical stromal invasion (9.59±4.87 vs. 7.47±5.48 mm, p = 0.048, Mann-Whitney U test), larger tumor size (2.32±1.15 vs. 1.74±1.14 cm, p = 0.043, Mann-Whitney U test), higher incidences of LVSI (87.5% vs. 28.8%, p<0.001, chi-square test), and lymph node metastasis (68.8% vs. 10.8%, p<0.001, chi-square test)compared to the group without parametrial invasion. Patients with LVSI (15.7% (14/89) vs. 1.1% (2/187)) or lymph node metastasis (28.2% (11/39) vs. 2.1% (5/237)) had significantly higher incidences of parametrial invasion compared to those without LVSI or lymph node metastasis (p<0.001 for both, chi-square test).

**Table 2:** Clinico-pathologic characteristics of 276 women at stages IA2 and IB1 with or without parametrial invasion.

Parametrial invasion	No (n = 260)	Yes (n = 16)	p
**Age** (years) (Mean±SD)	48.95±9.91	55.94±9.49	0.005^a^
**Menopause**			0.057^b^
No	160	6	
Yes	100	10	
**Gravida** (Mean±SD)	3.69±1.88	3.93±1.39	0.56^a^
**Parity** (Mean±SD)	2.28±1.45	3.44±1.32	0.022^a^
**Histology**			
SCC	178	14	0.59^b^
ADC	63	2	
ASC	12	0	
NEC	4	0	
Others*	3	0	
**Stage**			
IA2	10	0	0.14^b^
IB1 ≤2 cm	164	7	
IB1>2 cm	86	9	
**Tumor size**	1.73±1.14	2.32±1.02	0.045^a^
**Depth of invasion** (mm)	7.59±5.55	9.81±4.98	0.046^a^
(Mean±SD)			
**LVSI**			
No	185	2	<0.001^b^
Yes	75	14	
**Lymph node metastasis**			
No	232	11	<0.001^b^
Yes	28	5	

SD: standard deviation, SCC: squamous cell carcinoma, ADC: adenocarcinoma, ASC: adenosquamous carcinoma, NEC: neuroendocrine carcinoma

*: including two adenocarcinoma with focal neuroendocrine differentiation, two clear cell carcinoma, and one undifferentiated carcinoma

^a^: by Mann-Whitney U test

^b^: by Chi-square test.

### Stage IB1 patients with tumors ≤2 cm with lymph node metastasis had a high incidence of parametrial invasion

**Fig 1:**
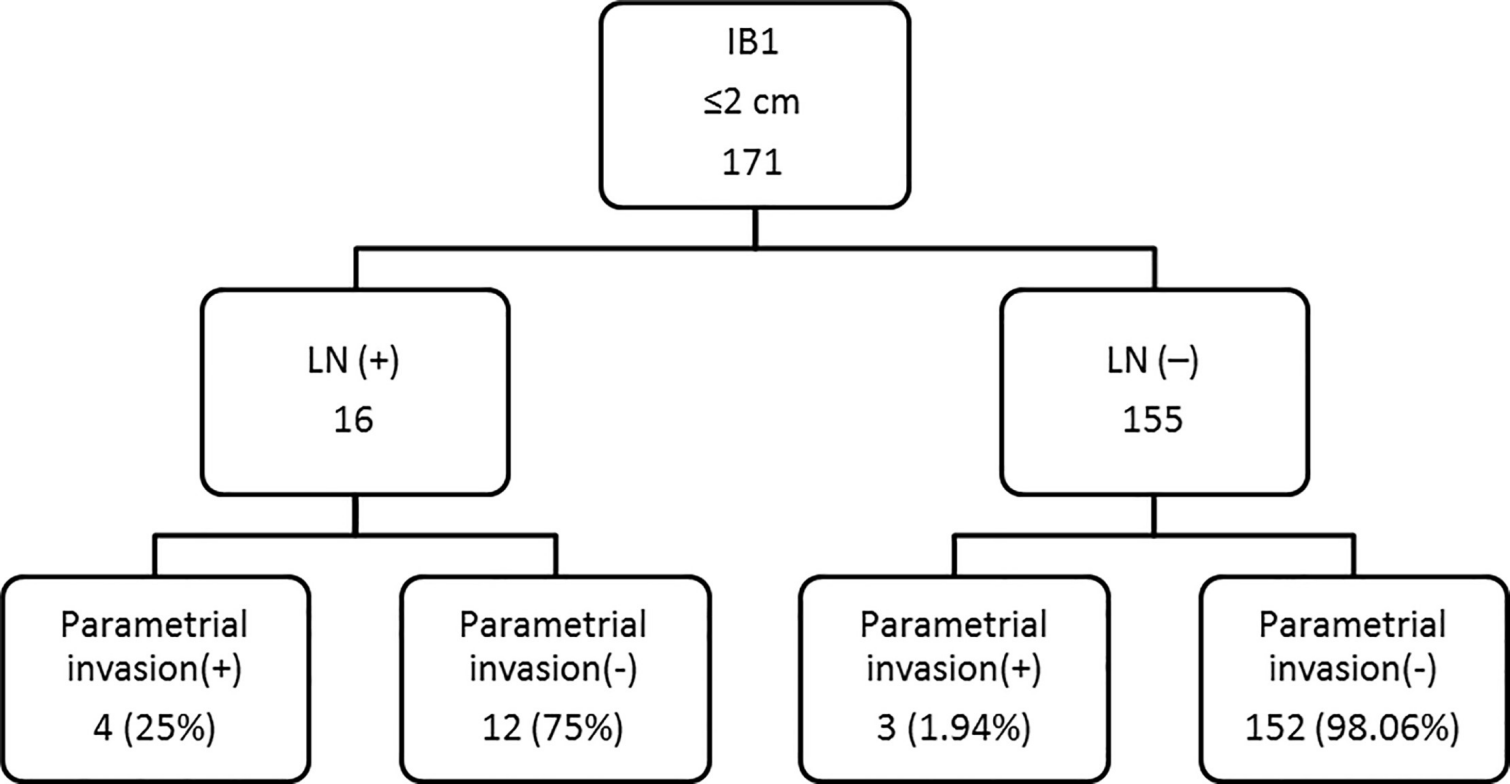
Scenario one to determine the need for parametrectomy during hysterectomy, according to the status of pelvic lymph node metastasis.

After stratification of the patients with stage IB1 tumors ≤2 cm into 2 groups with or without lymph node metastasis (Fig 1), 25% (4/16) of patients with lymph node metastasis had parametrial invasion, whereas it occurred only 3 of the 155 patients (1.9%) with negative lymph nodes. Patients with lymph node metastasis showed a higher overall incidence of parametrial invasion (25% vs. 1.9%, p<0.001, chi-square test).

### Characteristics of the stage IB1 patients withtumors ≤2 cm and parametrial invasion

The characteristics of the 7 patients with stage IB1 tumors ≤2 cm who had parametrial invasion are shown in Table 3. All 7 of these patients were aged older than 50 years (range 50–73), and there were 6 cases of squamous cell carcinomas and 1 adenocarcinoma. The tumor size ranged from 0.5 to 2 cm, and the depth of cervical stromal invasion (DOI) ranged from 5–12 mm. Five of these patients had LVSI, and 4 had lymph node metastasis. Three patients without lymph node metastasis but with parametrial invasion were aged older than 60 years. The other 4 patients were aged from 50 to 58 years and had both lymph node metastasis and parametrial invasion.

**Table 3:** Clinico-pathologic characteristics of 7 stage IB1 patients (tumor size ≤2 cm) with parametrial invasion.

Case	1	2	3	4	5	6	7
Age	63	60	73	51	54	50	58
Gravida	5	2	5	5	3	2	6
Para	4	2	5	4	3	2	4
Menopause	Yes	Yes	Yes	No	No	No	Yes
Histologic type	SCC	SCC	SCC	SCC	SCC	ADC	SCC
Tumor size (cm)	2	0.5	0.6	1.5	1.3	2	0.6
DOI (mm)	12	10	6	5	9	12	6
LVSI	Yes	Yes	No	No	Yes	Yes	Yes
Number of lymph node dissections	33	16	21	20	19	17	31
Number of lymph node metastases	0	0	0	1	6	6	1

SCC: squamous cell carcinoma, ADC: adenocarcinoma, DOI: depth of cervical stromal invasion, LVSI: lymphovascular space involvement

### Stage IB1 patients with tumors ≤2 cm aged younger than 50 years old had a rare rate of parametrial invasion

**Fig 2:**
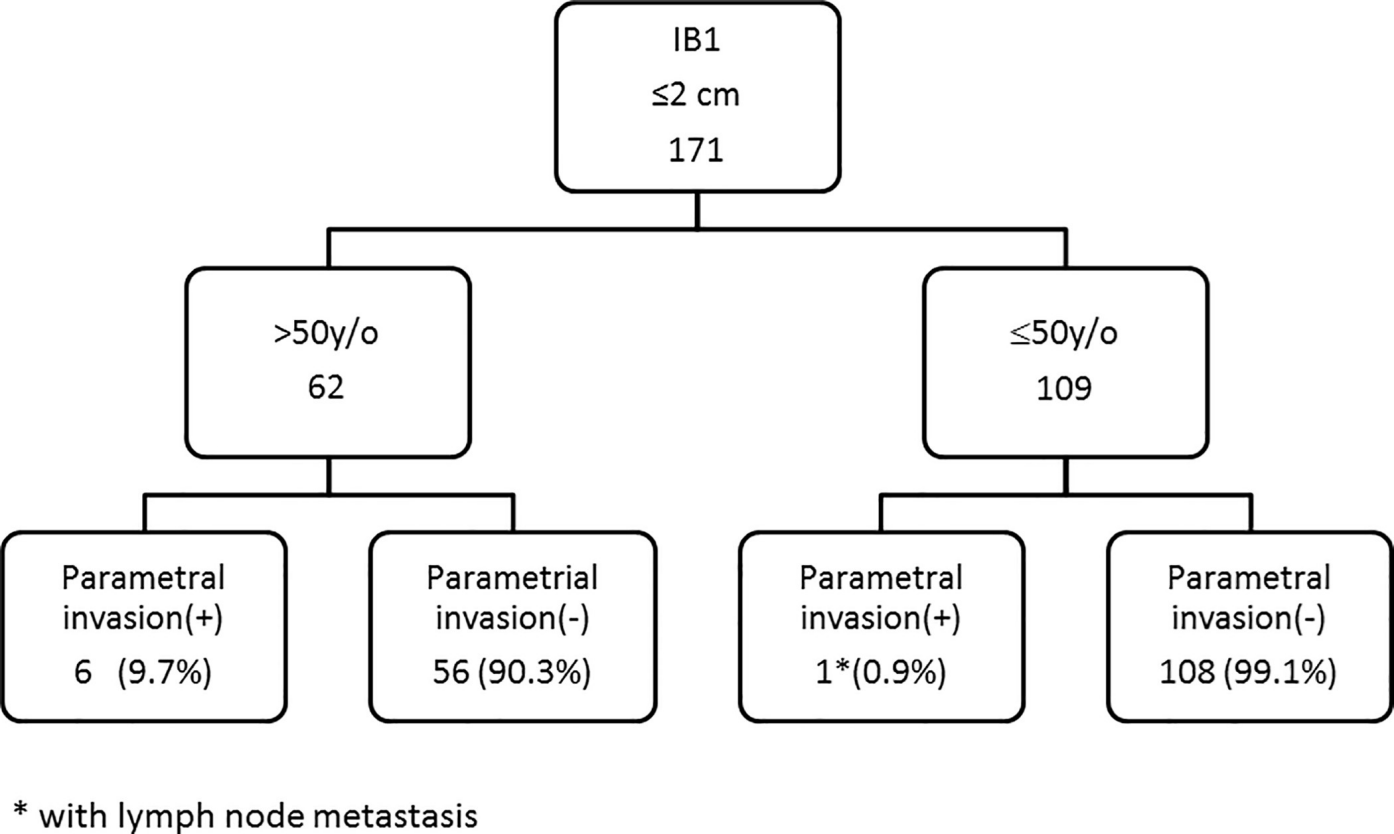
Scenario two to determine the need for parametrectomy during hysterectomy, according to patient age.

The stratification by age for 171 patients with stage IB1 and tumors ≤2 cm with or without parametrial invasion is shown in Fig 2.Patients aged over 50 years had a higher incidence of parametrial invasion compared to those aged less than 50 years (9.7% vs. 0.9%, p = 0.005, chi-square test). Only 1 in109 patients aged ≤ 50years had parametrial invasion, and this patient also had lymph node metastasis. In contrast, 6 (9.7%) of the 62 patients aged more than 50years had parametrial invasion.

## Discussion

Cervical cancer spreads locally, traversing through the lymphatic route and the parametrium to the pelvic sidewall. Therefore, the approach is to treat the disease with enbloc radical surgery including parametrectomy with negative surgical margins. However, there is an abundance of nerves in the parametrium. A parametrectomy can damage the hypogastric nerve, which results in colorectal disturbances, voiding dysfunction, and deterioration of sexual life [7]. Bowel dysfunction was noted in one-fifth of patients [21, 22] and fecal incontinence in one-third of patients undergoing radical surgery [22]. In addition, one-fourth of patients had incomplete emptying of the bladder and strenuous micturition [21, 23], while 40% had long-term voiding dysfunction [23, 24]. Clearly, decreasing the morbidities of hypogastric nerve injury is an important issue.

Nerve-sparing radical hysterectomy is an alternative surgical procedure developed to decrease hypogastric nerve injury-related morbidities. In the 1960s, a group of Japanese surgeons proposed methods to preserve the autonomic nerve fibers during radical hysterectomy [25, 26]. Sakuragiet al. reported that there was no incidence of incontinence and only 2 patients with a sensitive bladder out of 22 patients who successfully underwent a nerve-sparing radical hysterectomy [27]. Fujiiet al. also reported on 24 patients who underwent a nerve-sparing radical hysterectomy, and all had complete urination function within 21 days after surgery [26]. Ditto et al. reported that there was no significant difference in 5-year disease survival (79.8% vs. 78.9%) and overall survival (84.1% vs. 90.8%) between conventional radical hysterectomy and nerve-sparing radical hysterectomy [28]. However, there was less bladder dysfunction after nerve-sparing surgeries [28]. A recent meta-analysis showed that nerve-sparing radical hysterectomy required a longer operation time, and no prospective trials have compared the outcome of nerve-sparing surgery with conventional radical hysterectomy [29].

Avoiding an unnecessary parametrectomy is another way to decrease hypogastric nerve injury-related morbidities. Several studies have reported the treatment outcomes from non-radical surgery in early cervical cancer [30–34]. A study by Reade et al. showed a recurrence rate of only 4.5% (21/467), and 1.25% (6/479) died from the disease [15]. Landoni et al. compared class I and III hysterectomies in patients with tumor sizes ranging from 2–4 cm and found that15 patients had recurrence and 4 patients died from the disease in the class I hysterectomy group [33]. However, the rates of surgical morbidity, including urinary dysfunction, blood loss, and bowel dysfunction, were significantly decreased in the class I hysterectomy group [35].

Age is an important factor to predict parametrial invasion of early-stage cervical cancer. Kodama et al. observed that elderly cervical cancer patients more commonly have parametrial invasion [11]. No reports have thoroughly explained the reason for this, and we believe it is because the uterus and cervix become smaller after menopause [36].Consequently, if the cancer spreads in a similar fashion for all ages, there is a higher possibility of invasion of the parametrium owing to the smaller size of the atrophic and aged cervix.

Lymph node metastasis is another critical factor in predicting parametrial invasion. In the context of lymph node metastasis, the incidences of parametrial invasion ranged from 36.4 to 48% [35, 37]. Pluta et al. demonstrated that patients with early cervical cancer and a positive sentinel lymph node had a 28% incidence of parametrial invasion [38].Our series showed a parametrial invasion incidence of 25% (4/16) in the patients with tumors <2 cm and lymph node metastasis. However, the status of lymph node metastasis is not easily evaluated before a hysterectomy in early cervical cancer patients in order to determine whether or not to perform a parametrectomy.

Other factors such as LVSI, tumor size, or the DOI are also associated with parametrial invasion. Several investigators suggested that LVSI may be a predictor of parametrial invasion [37, 39], and it was reported that 30–60% of patients with stage I cervical cancer had LVSI. Although LVSI was significantly associated with parametrial invasion (p<0.001, chi-square test) in the present study, it is often not documented in the cervical biopsy or the conization specimens [39, 40]. Therefore, LVSI might not be a clinically useful predictor of parametrial invasion in early-stage cervical cancer.

The limitations of this study include its retrospective design with a variable follow-up duration and the lack of a pathologic review. The prospective trials of early cervical cancer patients undergoing non-radical surgery, including the SHAPE, GOG278, and ConCerv trials, are still ongoing. Their results may indicate that patients with early-stage cervical cancer could benefit from non-radical hysterectomy. Whether it is conventional or nerve sparing, a radical hysterectomy requires well-trained, experienced, and skillful surgeons. With the promotion of the Pap smear and HPV vaccination, the incidence of cervical cancer can be expected to steadily decrease. Thus, it is important to identify suitable patients for treatment with non-radical surgery, which is the value of this retrospective study.

Based on our results, we developed a possible algorithm to facilitate the selection of those cervical cancer patients for whom a parametrectomy is indicated during a hysterectomy. For stage IA2 patients, parametrectomy may be omitted. Parametrectomy should be performed for all stage IB1 patients with a tumor size >2 cm.

For stage IB1 patients with a tumor size ≤2 cm, we proposed two scenarios to determine whether parametrectomy is indicated using either the frozen pathologic sections of the pelvic lymph nodes or the patient’s age. For scenario one, as shown in Fig 1, since patients with lymph node metastasis (25% (4/16) vs. 1.9% (3/155), p<0.001,chi-square test) had a higher incidence of parametrial invasion compared to those without, we recommend sentinel lymph node sampling with frozen pathology before undergoing hysterectomy. If the frozen pathology of the lymph nodes ispositive, then a radical hysterectomy should either be performed with parametrectomy or abandoned due to the high incidence of parametrial invasion. If the frozen pathology of the lymph nodes is negative, then a simple hysterectomy without parametrectomy can be considered due to the low incidence of parametrial invasion.

For scenario two, we recommend radical hysterectomy for all patients aged over 50 years in hospitals without the resources to perform sentinel lymph node sampling or frozen pathology of lymph nodes. As shown in Fig 2, only 1 (0.9%) out of 109 of the patients aged ≤50 years had parametrial invasion, whereas 9.7% (6/62) of the patients aged over 50years had parametrial invasion. Thus, for stage IB1 patients with a tumor size <2 cm, parametrectomy can be omitted for patients aged ≤50 years, but radical hysterectomy still needs to be undergone for those aged over 50 years.

## Supporting information

S1 Table Limited raw data are included in the following file: 20180611. (XLS)
